# Etrasimod Treatment Modulates Circulating and Lymph Node-Derived Lymphocytes in Crohn’s Disease

**DOI:** 10.3390/ijms27052447

**Published:** 2026-03-06

**Authors:** Dimitrios Nikolakis, Maarten J. Pruijt, Jan Verhoeff, Floris A. E. de Voogd, Christoph Teichert, Rathi D. Ryan, Diogo Branquinho, Catherine Crosby, Marleen G. H. van de Sande, Joep Grootjans, Geert R. D’Haens

**Affiliations:** 1 Department of Gastroenterology and Hepatology, Amsterdam UMC, De Boelelaan 1117, 1081 HV Amsterdam, The Netherlands; d.nikolakis@amsterdamumc.nl (D.N.); m.j.pruijt@amsterdamumc.nl (M.J.P.); j.verhoeff@amsterdamumc.nl (J.V.); f.a.devoogd@amsterdamumc.nl (F.A.E.d.V.); c.teichert@amsterdamumc.nl (C.T.); j.grootjans@amsterdamumc.nl (J.G.); 2 Department of Rheumatology & Clinical Immunology, Amsterdam Rheumatology & Immunology Center (ARC), Amsterdam UMC, 1081 HV Amsterdam, The Netherlands; m.g.vandesande@amsterdamumc.nl; 3 Amsterdam Gastroenterology Endocrinology & Metabolism Institute, 1105 AZ Amsterdam, The Netherlands; 4 Department of Experimental Immunology, Amsterdam Institute for Immunology and Infectious Diseases, Amsterdam UMC, University of Amsterdam, Meibergdreef 9, 1105 AZ Amsterdam, The Netherlands; 5 Center for Infectious Medicine (CIM), Department of Medicine Huddinge, Karolinska Institutet, ANA Futura, 141 52 Huddinge, Sweden; 6 Pfizer Inc., South San Francisco, CA 94080, USA; rathi.ryan@pfizer.com; 7 Pfizer Inc., New York, NY 10001, USA; diogo.ferreirabranquinho@pfizer.com; 8 Pfizer Inc., San Diego, CA 92121, USA; catie.crosby@pfizer.com

**Keywords:** Crohn’s disease, etrasimod, S1P receptor, lymphocytes, lymph nodes

## Abstract

Etrasimod is an oral selective sphingosine-1 phosphate receptor modulator, and its anti-inflammatory mechanism of action in inflammatory bowel diseases is not completely understood. It targets pro-inflammatory immune cells expressing sphingosine-1-phosphate receptors during their migration from the lymphatic system to the circulation and intestinal mucosa. Reductions in certain lymphocyte subsets in the peripheral blood have been reported, but its effects in lymph nodes remain unknown. This study investigated changes in leukocyte subpopulations in peripheral lymph nodes and blood in Crohn’s disease patients treated with etrasimod. Moderate-to-severe Crohn’s disease patients participated in this randomized, double-blind study, within the phase 2 CULTIVATE clinical trial. At baseline and after 14 weeks of etrasimod treatment, peripheral blood and inguinal lymph node biopsies were obtained. Isolated peripheral blood mononuclear cells and lymph node leukocyte populations were analyzed at single cell level using mass cytometry at both timepoints. The immunophenotyping revealed 15 innate and adaptive major immune cell populations, as well as 14 subpopulations of CD4+ and CD8+ T-cells. In peripheral lymph nodes, etrasimod resulted in significant accumulation of naïve, central memory, and effector memory CD4+ T-cells (+10.7%, +4.2%, and +2.3%, respectively; all *p* = 0.03), as well as naïve CD8+ T-cells (+4.2%; *p* = 0.03). Conversely, these subsets were reduced in peripheral blood (−6.2%, −6.0%, −2.0%, and −2.2%, respectively; all *p* = 0.03). Naïve and memory B-cells decreased in the circulation (−1.7%, *p* = 0.057; −0.6%, *p* = 0.03, respectively) but were unchanged in the lymph nodes. Innate immune cell populations remained mostly unaffected in both compartments. Our data indicate that etrasimod’s pharmacodynamic effect is related primarily with the attenuation of the T-cell mediated inflammation with minor changes in B-cells. However, additional follow-up studies are needed for the validation of these observations in the context of Crohn’s disease.

## 1. Introduction

Inflammatory bowel diseases (IBD) such as Crohn’s disease (CD) and ulcerative colitis (UC) are chronic immune-mediated diseases of the gut causing symptoms such as abdominal pain and diarrhea [1,2]. While the exact cause of IBD remains largely unknown, previous research suggests that it stems from an abnormal immune response to the gut microbiome, influenced by both genetic and environmental factors [3]. As part of the pathophysiology, the adhesion of lymphocytes on the intestinal endothelium is followed by migration of these cells to the inflamed mucosa and submucosa, perpetuating chronic inflammation [1,2,4]. Microbial components that penetrate the luminal side are recognized by dendritic cells, which then migrate to local lymphoid tissues, where they present these antigens to naïve T-cells [2,3]. This process leads to the activation and differentiation of these cells into specific T-helper subtypes under the influence of cytokines. Subsequently, these cells leave the lymph nodes, re-enter the circulation via the lymphatics, and return to inflamed tissues, amplifying the cellular immune response [3,4,5,6]. The migration of lymphocytes into the intestinal mucosa is a key factor in the development of chronic inflammation in IBD [5]. Other lymphocyte types, including B-cells and natural killer (NK) cells, also contribute to IBD pathogenesis [6]. Therefore, inhibition of lymphocytes egression from lymphoid tissues into the systemic circulation and consequently to the intestinal mucosa holds promise as a therapeutic approach in IBD [5,7].

One way to achieve this effect is by targeting the receptor of a bioactive sphingolipid called sphingosine-1-phosphate receptor (S1PR) [7]. In the lymph nodes (LNs), there is a gradient of the sphingosine-1-phosphate (S1P) molecule from the afferent towards the efferent lymphatic vessels, with the highest concentrations emerging at the efferent lymphatic vessels [7]. By binding to S1PRs, the S1P molecule acts as a chemoattractant for immune cells, such as lymphocytes, towards the LN areas that this sphingolipid is present in higher concentrations, subsequently leading to the migration of these cells to other tissues. The mechanism of action of S1PR modulators, such as etrasimod, is based on the internalization of S1P receptors in lymphocytes, hence causing them to lose responsiveness to S1P molecules and inhibiting their trafficking by retaining them within secondary lymphoid organs, such as the LNs [8]. Such retention results in a reduction of circulating lymphocyte transmigration to the sites of inflammation.

Etrasimod has already been approved for the treatment of moderately to severely active UC [9] and is currently under investigation for the treatment of CD. Previous studies involving peripheral blood samples of healthy individuals and atopic dermatitis patients receiving etrasimod demonstrated a reduction in naïve and central memory T-helper cells, as well as in Th2 cells and at a lesser extent in cytotoxic T-cells, while the results regarding B-lymphocytes were contradictory, displaying either a reduction or no effect [8]. Additionally, in the atopic dermatitis patients, skin-homing T-cells were reduced, while monocytes, conventional dendritic cells and NK-cells were upregulated after etrasimod treatment [8]. Based on these findings, we hypothesize that etrasimod treatment primarily affects the T-lymphocytes, compared to other innate and adaptive immune cell phenotypes.

Even though the effects of S1PR modulators on circulating immune cells have been previously documented [10], immune cell changes within LNs, associated with etrasimod treatment, have not been characterized in the context of human studies. LNs represent the primary anatomical site where lymphocytes undergo antigen-driven activation, differentiation, and subsequent S1P-dependent egress into the circulation [7,8]. Based on animal models, the main target tissue of S1PR blockers is the LN compartment [7]; however, there are no human data to confirm this. This fact indicates a gap of knowledge concerning the molecular mechanism of these compounds, especially in IBD. Consequently, the characterization of immune cell dynamics within LNs is essential to mechanistically validate altered trafficking patterns observed in PB. Paired immunophenotypic analysis of LN and PB samples provides a more comprehensive understanding of how etrasimod reshapes immune cell distribution. Therefore, the aim of this study was to investigate alterations in the leukocyte subpopulations within the lymphatic system (inguinal LNs) and the PB in response to etrasimod treatment in patients with CD, to further elucidate the drug’s pharmacodynamic effect at the cellular and molecular level.

## 2. Results

**Figure 1:**
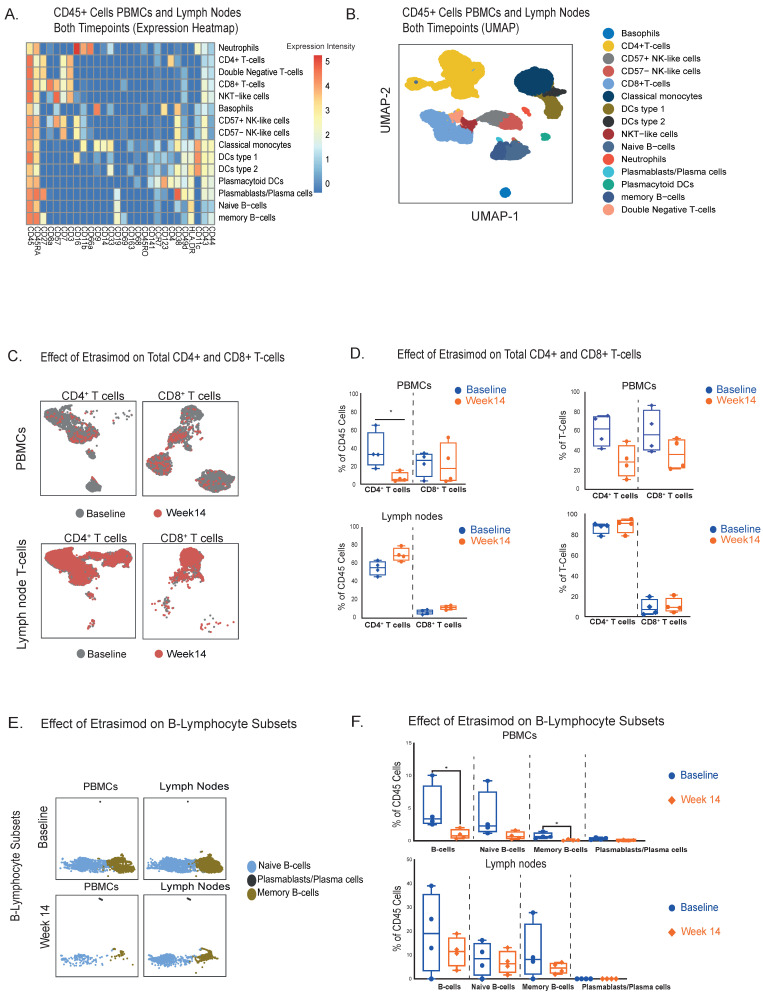
(**A**). Immunophenotyping heatmap of all leukocytes at both compartments (PB and LNs) and timepoints of etrasimod treatment (Baseline and Week 14), based on the relative expression of specific protein markers (*n* = 4 patients). (**B**). UMAP visualization of the different clusters of lymphocytes and innate cells at both compartments. (**C**). UMAP visualization of the total CD4+ and CD8+ T-cell clusters at both compartments and timepoints. (**D**). Quantification of differences of the CD4+ and CD8+ T-cells between baseline and week 14 in PBMCs and LNs. Total CD4+ T-cells were reduced in PB (*Median % Difference from Baseline: −22.0% [95%CI: −48.4 to −11.1], p = 0.03*)/(*Median % Difference of total T-cells from Baseline: −33.9% [95%CI: −47.8 to −20], p = 0.057*) and increased in the LNs (*Median % Difference from Baseline: +13.2% [95%CI: +5.9 to +34.2], p = 0.057)/(Median % Difference of total T-cells from Baseline: +2.3% [95%CI: +0.3 to +4.7], p = 0.2*). (**E**). UMAP visualization of B-cell subsets at both compartments and timepoints (cell counts are depicted in the UMAP). (**F**). Quantification of differences in B-cell subpopulations between baseline and week 14 in PBMCs and LNs. In PB significant reductions were observed in total B-cells and naïve B-cells (*Median % Difference from Baseline: −2.6% [95%CI: −9.5 to −0.5], p = 0.03 and −0.6%, [95%CI: −1.3 to −0.08], p = 0.03, respectively*), followed by a decrease in naïve B-cells (*Median % Difference from Baseline: −1.7% [ 95%CI: −8.8 to −0.4], p = 0.057*), while plasmablasts were slightly attenuated (*Median % Difference from Baseline: −0.2% [95%CI: −0.4 to 0.0], p = 0.14*). In LNs there were no significant changes in naïve and memory B-cells (*Median % Difference from Baseline: −7.5% [95%CI: −35.4 to +10.5], p = 0.47, −2.2% [95%CI: −9.6 to +7.3], p = 0.89 and −3.6% [95%CI: −3.3 to +25.9], p = 0.34, respectively*), as well as in plasmablasts (*Median % Difference from Baseline: −0.01% [95%CI: −0.02 to +0.02], p = 0.99*). * *p*-value < 0.05.

High-dimensional CyTOF-aided immunophenotyping identified 15 innate and adaptive immune cell populations detectable in both PB and LN samples at baseline and after 14 weeks of etrasimod induction therapy (Figure 1A,B).

### 2.1. CD4+ and CD8+ T-Lymphocytes Are Redistributed from Peripheral Blood to LNs During Etrasimod Treatment

As a next step we focused on the total CD4+ and CD8+ T-cell populations at both timepoints (Baseline and Week 14) (Figure 1C). Our analysis revealed a significant reduction of circulating CD4+ T-cells, 14 weeks after the initiation of etrasimod treatment (Figure 1D). Conversely, we observed nearly significantly increased levels of CD4+ T-cells in LNs. CD8+ T-cells displayed the same trend in both compartments, though the differences were not significant (Figure 1D). Similar trends with no statistically significant differences were also displayed, when the quantification of CD4+ and CD8+ T-cells was expressed as a percentage of the total T-cells (Figure 1D). For instance, CD4+ T-cells were nearly significantly reduced in PB at week 14, while they were slightly increased in the LNs. CD8+ T-cells displayed the same pattern with a not significant decrease in the PB and a not significant increase in the LNs. Collectively, CD4+ T-cells were the ones mostly affected by etrasimod treatment, displaying an increase in the LN compartment and a respective reduction in PB. A similar pattern was also observed in CD8+ T-cells, but to a lesser extent than T-helper lymphocytes.

### 2.2. Etrasimod Treatment Results in a Reduction of Circulating Naïve and Memory B-Cells, Accompanied by an Increase in Circulating Dendritic Cells and Classical Monocytes, Without a Significant Impact in LNs

Using our CyTOF panel, we profiled three different B-cell clusters: naïve B-cells (CD19+, CD27−, CD38−), plasmablasts (CD19+, CD27+, CD38+) and memory B-cells (CD19+, CD27+, CD38−) (Figure 1A). B-cell subpopulations overall appeared to be decreased in the circulation and not significantly altered in the LNs (Figure 1E). Specifically, we detected significant reductions of total circulating B-cells and circulating memory B-cells 14 weeks after the etrasimod treatment initiation, and a nearly significant decrease in naïve B-cells. Plasmablasts were not significantly decreased (Figure 1F).

In the LNs, we did not observe significant changes in total, naïve and memory B-cells (Figure 1F). The LN plasmablasts counts were low, and there were no notable differences in this cell population after etrasimod exposure (Figure 1F).

**Figure 2:**
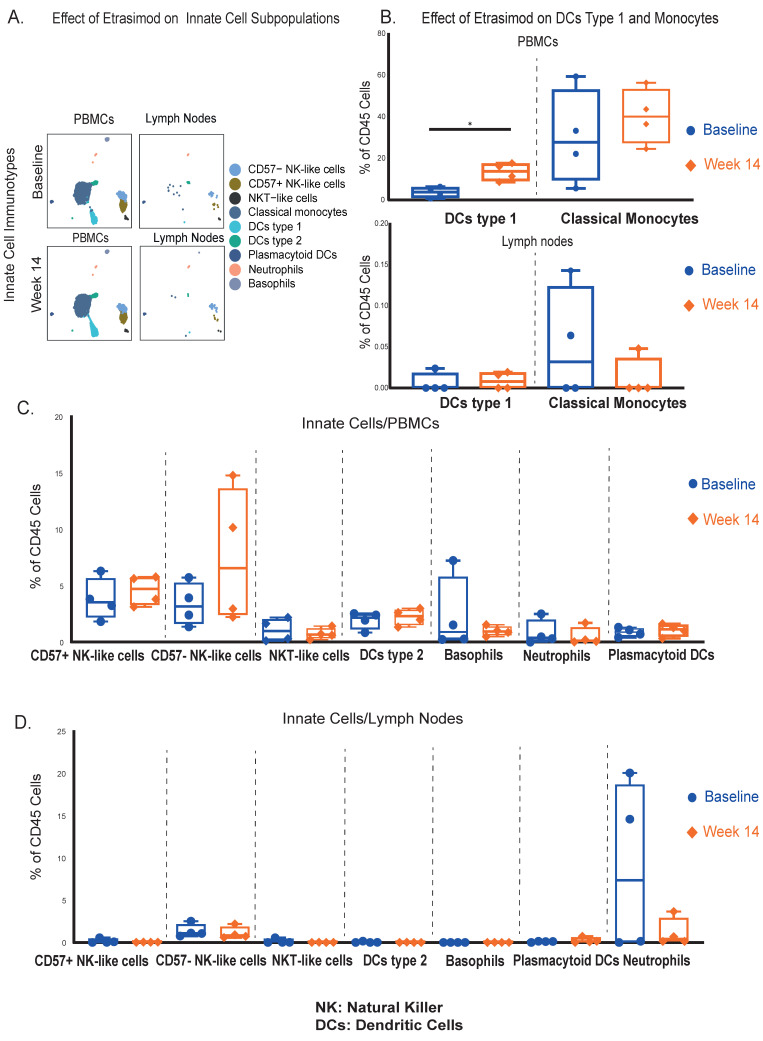
(**A**). UMAP cluster visualization of all the innate immunotypes at both compartments and timepoints (cell counts are shown in the UMAP). (**B**). Quantification of differences in the dendritic cells type 1 and classical monocytes, between baseline and week 14 in PBMCs and LNs. In PBMCs, conventional DCs type 1 were significantly increased (*Median % Difference from Baseline: +10.0% [95%CI: +5.9 to +15.4], p = 0.03*), while classical monocytes were also upregulated, without reaching statistical significance (*Median % Difference from Baseline: +12.4% [95%CI: −38.1 to +34.8], p = 0.49*). In the LNs, both cell populations remained unaffected. (**C**). Quantification of differences between the 2 timepoints, in additional phenotyped innate cell populations in PBMCs. (**D**). Quantification of differences between the 2 timepoints, in additional phenotyped innate cell populations in LNs. * *p*-value < 0.05.

Concerning innate leukocytes (Figure 2A), circulating conventional dendritic cells type 1 (DCs type 1) (CD11c+, CD141+, HLA-DR+) were significantly increased at week 14, while no significant differences were observed in LNs (Figure 2Β). Classical monocytes (CD14+, CD11b+, CD16−, HLA-DR+) were slightly increased in the circulation (Figure 2Β) and remained unaltered within the LNs (Figure 2Β). Other circulating (Figure 2C) and LN innate cell types (Figure 2D) did not display significant differences between the 2 timepoints.

The abovementioned findings highlight the selective impact of etrasimod on adaptive immune cells, with notable alterations primarily in T-cell populations followed by B-cell subsets, while innate immune cells were not significantly affected.

### 2.3. T-Cell Subphenotypes, Including Naïve, Central Memory and Effector Memory T-Cells Are Reduced in the PB and Increased in the LNs by Etrasimod

**Figure 3:**
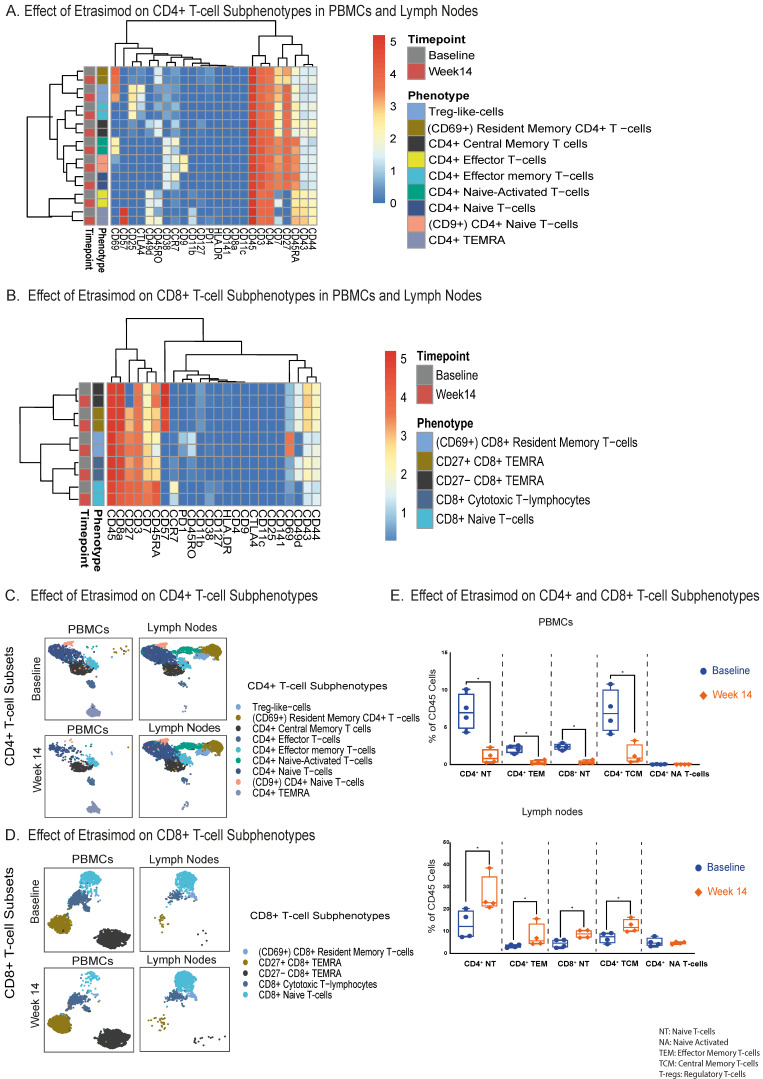

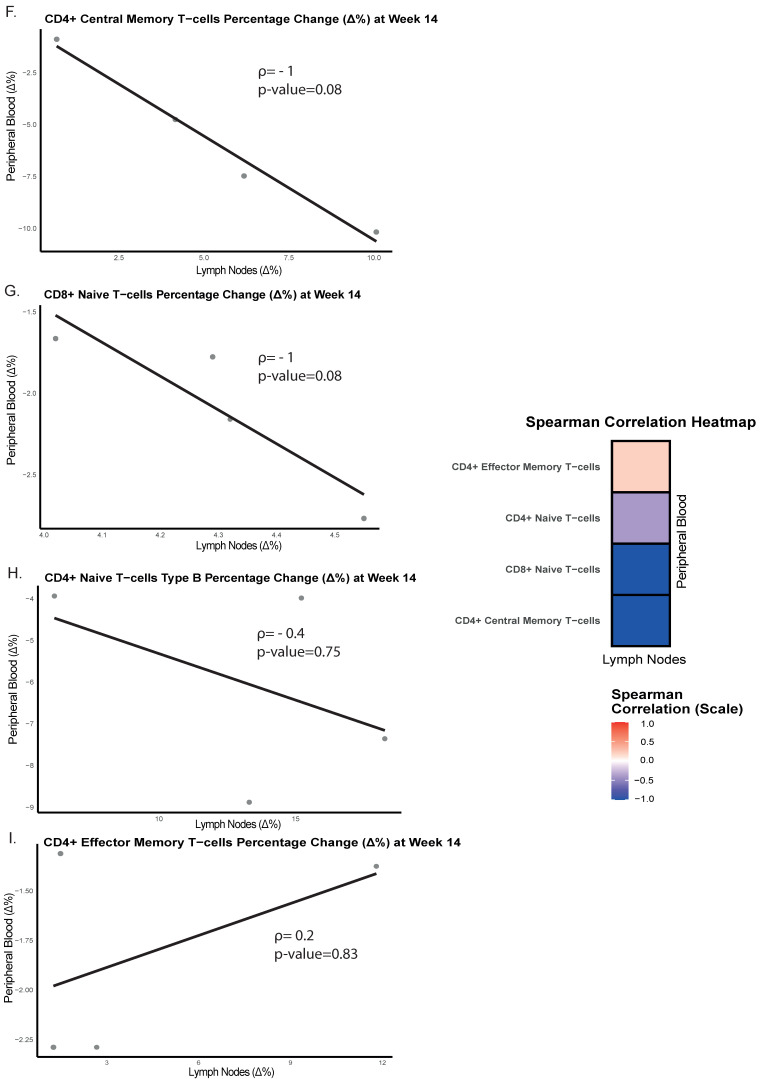
(**A**). Immunophenotyping heatmap, representing the reclustering on the CD4+ T-cell subpopulations, based on the relative expression of specific cellular protein markers at both compartments and timepoints (baseline and week 14). (**B**). Immunophenotyping heatmap, representing the reclustering on the CD8+ T-cell subpopulations, based on the relative expression of specific cellular protein markers at both compartments and timepoints (baseline and week 14). (**C**). UMAP cluster visualization of the CD4+ T-cell subsets at both compartments and timepoints (cell counts are depicted in the UMAP). (**D**). UMAP cluster visualization of the CD8+ T-cell subsets at both compartments and timepoints (cell counts are depicted in the UMAP). (**E**). Quantification of the most significant differences (*p* < 0.05) of the CD4+ and CD8+ T-cell subsets between baseline and week 14 in PBMCs and LNs. In PBMCs at week 14, statistically significant reductions were observed in CD4+ naïve *(Median % Difference from Baseline: −6.2% [95%CI: −8.9 to −3.9], p = 0.03*), CD4+ central memory (*Median % Difference from Baseline: −6.0% [95%CI: −10.2 to −0.9], p = 0.03*), CD4+ effector memory (*Median % Difference from Baseline: +2.3% [95%CI: +1.6 to +11.7], p = 0.03*), and CD8+ naïve T-cells (*Median % Difference from Baseline:−2.2% [95%CI: −2.8 to −1.7], p = 0.03*). In the LNs at week 14, statistically significant accumulations were detected in CD4+ naïve (*Median % Difference from Baseline: +10.7% [95%CI: +6.2 to +18.3], p = 0.03*), CD4+ central memory (*Median % Difference from Baseline: +4.2% [95%CI: +0.7 to +10.1], p = 0.03*), CD4+ effector memory (*Median % Difference from Baseline: +2.3% [95%CI: +1.6 to +11.7], p = 0.03*) and in the CD8+ naïve T- lymphocytes (*Median % Difference from Baseline: +4.2% [95%CI: +4.0 to +4.6], p = 0.03*). (**F**). Spearman correlation plots (including a Spearman correlation heatmap), between lymph nodes and peripheral blood, comparing percentage differences from baseline until week 14 of etrasimod treatment in CD4+ central memory T-cells, (**G**). CD8+ naïve T-cells, (**H**). CD4+ naïve T-cells, and (**I**). CD4+ effector memory T-cells. * *p*-value < 0.05.

As etrasimod treatment resulted in significant changes in the T-lymphocyte populations at both PB and LNs, we next analyzed subpopulations of CD4+ and CD8+ T-cells within these compartments to better elucidate which subsets are affected. Via our CyTOF panel, we profiled nine CD4+ T-cell subphenotypes (Figure 3A) and five CD8+ T-cell subphenotypes (Figure 3B). These cell clusters were also compared between the 2 timepoints (Figure 3C,D). Interestingly, we observed significantly reduced numbers of circulating CD4+ effector memory, CD4+ central memory, CD4+ naïve, and CD8+ naïve T-cells after 14 weeks of etrasimod treatment (Figure 3E). Other circulating CD4+ and CD8+ T-cell subsets, and double negative (DN) T-cells, showed no significant alterations after etrasimod treatment (Supplementary Figure S1).

Compared to baseline and in line with our previous observations in the PB, the proportion of the effector and naïve T-cells was elevated at week 14 after treatment, in the LNs. This observation was mostly pronounced in the CD4+ effector memory, the CD4+ central memory, the CD4+ naïve and the CD8+ naïve T-lymphocytes (Figure 3E).

Changes in the LN CD4+ naïve T-cells (*+10.7% median increase*) were more pronounced than in other T-cell subpopulations, in line with our findings in PBMCs (*−6.2% median reduction*). Etrasimod treatment did not significantly impact the remaining LN CD4+ and CD8+ T-cell subsets that were phenotyped, as well as the LN DN (CD4−, CD8−) T-cells (Supplementary Figure S1).

A correlation analysis between PB and LNs was performed to assess the relationship of the abovementioned T-lymphocyte changes induced by etrasimod treatment, within these two compartments. A strong negative correlation, although not statistically significant, was detected between LNs and PB regarding CD4+ central memory (*ρ = −1, p = 0.08*) (Figure 3F) and CD8+ naïve T-lymphocytes (*ρ = −1, p = 0.08*) at week 14 (Figure 3G). In CD4+ naïve T-cells (Figure 3H) a not significant negative correlation emerged (*ρ = −0.4, p = 0.75*), while CD4+ effector memory T-cells appeared to have a slight no clear positive correlation between LNs and PB (*ρ = 0.2, p = 0.83*) (Figure 3I).

### 2.4. CD69, CTLA-4 and PD-1 Expression Is Reduced in LN CD4+ T-Cell Subsets by Etrasimod

Based on our CyTOF analysis, immune cell activation and suppression status was also evaluated via the expression of representative markers such as CD69, CTLA-4 and PD-1 in lymphocyte populations that showed significant differences (total CD4+ and CD8+ T-cells, CD4+ naïve, central memory and effector memory T-cells and CD8+ naïve T-lymphocytes). Additionally, CD69 can have a pleiotropic effect as a tissue residency marker and also plays a role in the S1P pathway by interacting with specific S1PR sub-domains in lymphocytes [11].

**Figure 4:**
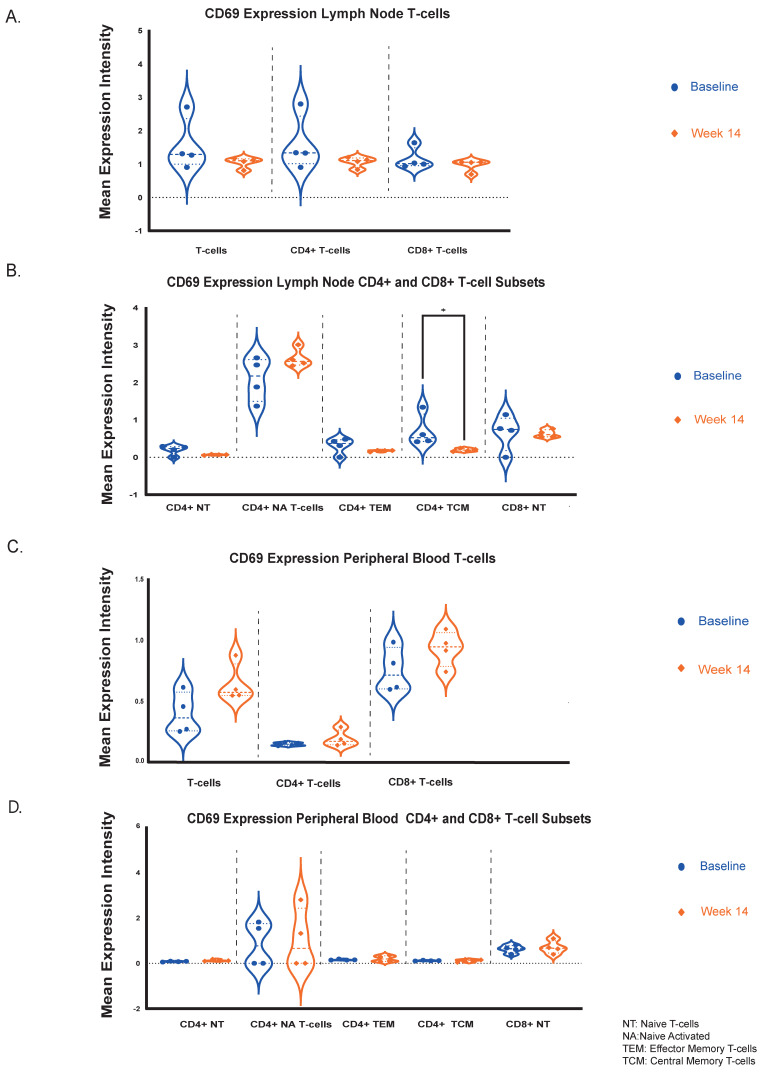
(**A**). Mean expression levels quantification of the CD69 protein marker in total T-lymphocytes, CD4+ T-cells and CD8+ T-cells at both timepoints (baseline and week 14) in the LNs. (**B**). Mean expression levels quantification of the CD69 protein marker at both timepoints, in the LN CD4+ and CD8+ T-cell subpopulations that were mostly affected by etrasimod. In the LNs, CD4+ central memory T-cells displayed a statistically significant reduction of CD69 expression levels (*Mean Expression Intensity Difference from Baseline = −0.5 [95%CI: −0.9 to −0.2], p = 0.029*). (**C**). Mean expression levels comparison of the CD69 protein between the 2 timepoints in total T-lymphocytes, CD4+ T-cells and CD8+ T-cells in PB. (**D**). Mean expression levels comparison of the CD69 protein between the 2 timepoints in the circulating CD4+ and CD8+ T-cell subpopulations that were mostly affected by etrasimod. * *p*-value < 0.05.

In LN-derived effector and naïve T-lymphocyte subsets, the mean expression levels of CD69 were numerically reduced after 14 weeks of treatment with etrasimod, but these differences were not statistically significant (Figure 4A,B). However, in the LNs, CD4+ central memory T-lymphocytes demonstrated significantly reduced CD69 expression levels at week 14 (*Mean Expression Intensity Difference from Baseline = −0.5 [95%CI: −0.9 to −0.2], p = 0.029*) (Figure 4B). On the contrary, in the PB, the CD69 expression levels of T-lymphocyte subsets remained unaltered (Figure 4C,D). CD69 expression was also not affected in the B-cell populations at both compartments and timepoints (Supplementary Figure S2A,B).

The immune-checkpoint protein CTLA-4 expression levels in the LNs were numerically but not statistically significantly reduced in total T-cells and CD4+ T-cells after 14 weeks of etrasimod treatment (Supplementary Figure S2C), whereas no significant changes were observed in PB (Supplementary Figure S2D). Similar results were observed concerning the expression levels of the exhaustion marker PD-1 in LN CD4+ T-cells, where a numerical but not statistically significant reduction was detected (Supplementary Figure S2Ε). Inversely, there was an increase of PD-1 expression in the circulating CD4+ T-cells (Supplementary Figure S2F). CTLA-4 and PD-1 expression in the CD8+ T-cells remained unaltered over time at both compartments (Supplementary Figure S2).

Overall, this analysis suggests that while the CD69 protein showed minimal changes in PB and LN lymphocytes, a notable reduction was observed specifically in the LN CD4+ central memory T-cells. CTLA-4 exhibited decreased expression in the LN CD4+ T-cells, but remained stable in the periphery while PD-1 levels in CD4+ T-cells were elevated in the PB and reduced in the LNs, though these changes were not statistically significant. Therefore, etrasimod potentially affects the activation status of T-helper cells as represented by CD69 expression, with differential effects between the PB and the LN compartments.

## 3. Discussion

This study provides, to our knowledge, the first comprehensive CyTOF-based assessment of an S1PR modulator on immune cell distributions and frequencies in both PB and LNs. Following 14 weeks of etrasimod therapy in CD patients, naïve, central memory, and effector memory CD4+ T-cells accumulated within the LNs, while they were downregulated in the circulation, indicating compartmental redistribution. These findings align with earlier flow cytometry data obtained from healthy volunteers treated with etrasimod [12], as well as with observations describing similar effects of other S1PR modulators across multiple indications [10,13,14,15,16,17,18,19]. In agreement with those studies, naïve and central memory subsets were the ones mostly affected, followed by effector memory T-helper cells, while total CD4+ T-cells were more responsive to treatment than CD8+ T-cells.

Innate immune cell populations remained largely unaltered, with the exception of the significantly upregulated type 1 DCs in PB at week 14 of etrasimod treatment. We can hypothesize that this observation can be explained by the potentially lower S1PR expression in innate cells, compared to lymphocytes at baseline. This notion though requires validation by future studies.

The inverse correlations between LN- and PB-derived T-cell subsets further support the interpretation that etrasimod primarily impedes lymphocyte egress rather than inducing depletion. Effector memory T-helper cells appeared less sensitive to the treatment, which is in line with previous clinical observations, where no significant changes in effector memory T-cells were observed in the PB [19]. However, no previously published data are available regarding their behavior in the LNs. B-cell responses were less consistent, since the substantial reduction of naïve and memory B-cells observed in PB was not accompanied by a corresponding significant accumulation in LNs, suggesting that B-cells may respond differently to S1PR modulation than T-cells.

While etrasimod primarily functions through modulation of lymphocyte trafficking via S1PR internalization, its effects on immune activation and retention markers appeared subset- and compartment-specific. CD69 plays a role in intestinal inflammation [20], while it also serves both as an early activation marker and as a regulator of lymphocyte retention through antagonism of S1P1 signaling [21,22]. CD69 protein levels remained stable across most lymphocyte populations, but not universally. A significant reduction was observed only in LN CD4+ central memory T-cells. Notably, the other T-cell subsets that were significantly affected by etrasimod treatment in the LN compartment—including CD4+ naïve, CD4+ effector memory, and CD8+ naïve T-cells—followed the same numerical trend of reduced CD69 expression, although these changes did not reach statistical significance. This consistent directional pattern suggests a potential mechanistic link between pharmacologic S1PR internalization and CD69-dependent lymphocyte retention dynamics. Previously published data demonstrated that the retention of lymphocytes in the LNs was achieved by reducing their responsiveness towards the S1P molecule [21,22]. In the LNs, the selective reduction of CD69 protein expression in central memory T-cells, and to a lesser extent in naïve and effector memory T-lymphocytes, possibly reflects a compensatory homeostatic feedback response attempting to restore migratory potential under sustained S1PR blockade. The inverse correlation between the S1P1R and CD69 expression was also supported by in vitro findings from a previous systemic lupus erythematosus trial assessing the pharmacologic effects of the S1P1R modulator cenerimod [23]; however, additional translational studies are essential to confirm this hypothesis.

In contrast to the CD69 expression data, the relative stability of CTLA-4 and PD-1 across most T-cell subsets supports the concept that etrasimod predominantly redistributes lymphocytes rather than inducing broad immunosuppressive or activation-altering effects. There was only a numerical but not statistically significant reduction of PD-1 and CTLA-4 expression in total LN CD4+ T-cells. PD-1 expression reflects both recent activation and sustained antigen exposure associated with T-cell exhaustion; therefore, its reduction may indicate decreased chronic antigenic stimulation within lymphoid tissues or redistribution of PD-1^high^ T-lymphocyte subsets rather than a direct attenuation of activation [24]. In contrast, CTLA-4 primarily regulates early activation thresholds and is also constitutively expressed on regulatory T-cells [24]. The observed numerical decrease in CTLA-4 may therefore reflect subtle shifts in activation dynamics or subset composition rather than exhaustion-related changes. Conversely, the mild increase in circulating PD-1 expression may represent relative enrichment of previously activated or exhausted T-cell subsets in the periphery following preferential retention of naïve and central memory populations within LNs.

Although S1P signaling can interface indirectly with immune activation pathways, S1PR modulators primarily influence chemotactic responsiveness rather than directly modulating intracellular activation cascades [7,8]. The largely unchanged expression of these checkpoint markers further suggests that immune activation states remain primarily governed by antigen exposure and local inflammatory cues, which may not be substantially altered during short-term trafficking modulation. Importantly, CD69 stability was most pronounced in T-cell populations that were not significantly affected by etrasimod in the context of LN homing, reinforcing its dual functional role as both an activation and retention marker. Overall, given the limited cohort size and absence of functional assays, these interpretations remain speculative and require further investigation.

While this study provides the first paired characterization of LN and PB immune modulation following S1PR targeting in CD, it is limited by the absence of mucosal immune profiling and a placebo-controlled comparator arm. The primary focus of this translational substudy was to elucidate lymphocyte trafficking dynamics within systemic and lymphoid compartments, a previously unexplored mechanistic aspect of etrasimod therapy in IBD. Although mucosal tissues represent the principal inflammatory site, the consistent redistribution of naïve and memory T-cell subsets between LNs and circulation that is observed across patients aligns with the established pharmacologic mechanism of S1PR modulation and is unlikely to reflect nonspecific disease fluctuation alone. However, immune cell dynamics within the mucosa may differ from systemic and lymphoid compartments, since they are shaped by distinct antigen exposure and tissue microenvironments [3,4,7]. Therefore, future longitudinal studies integrating mucosal biopsies, clinical endpoints, and controlled treatment arms will be essential to directly link altered trafficking patterns with local intestinal immune activity and therapeutic response.

This exploratory study is also limited by its small sample size (*n* = 4), which may disproportionately affect the detection of subtle changes in low-frequency immune populations and limits stratified analyses accounting for inter-patient variability in disease severity and treatment history. Nevertheless, the consistent directional modulation observed across key T-cell subsets supports a dominant pharmacodynamic effect of etrasimod on lymphocyte trafficking.

## 4. Materials and Methods

### 4.1. Patient Material

Adults with moderate-to-severe CD who exhibited insufficient response or loss of response to at least one prior therapy and participated in the phase 2, double-blind, non-placebo-controlled CULTIVATE trial (SSA-P2; EudraCT: 2020-004775-40) were eligible for this translational substudy. Four patients were included. Baseline characteristics are summarized in Table 1.

Participants were randomized to receive etrasimod 2 or 3 mg daily. Clinical assessment, including CDAI scoring and biomarker measurements (CRP and FCP), was performed at baseline and week 14. In addition to study procedures, peripheral blood samples (10 cc) and four ultrasound-guided inguinal LN core-needle biopsies were obtained at both timepoints. The biopsy procedure has been described previously [25]. Peripheral blood mononuclear cells (PBMCs) and LN leukocytes were isolated for downstream analysis.

### 4.2. Cytometry by Time-of-Flight (CyTOF)

Single-cell immunophenotyping was conducted via mass cytometry (CyTOF) to characterize immune cell composition. LN and PBMC cell suspensions were barcoded using palladium isotopes according to the manufacturer’s protocol (Fluidigm/standard biotools, standardized kit protocol, catalogue number: PN201060, Markham, ON, Canada). The samples were pooled together for further analysis, to minimize potential batch effects.

A panel of 36 metal-conjugated antibodies targeting innate, B-cell, and T-cell markers was applied (Supplementary Table S1). Data acquisition was performed on a Helios™ mass cytometer. After quality control and removal of cellular debris and non-viable cells, unsupervised cell clustering and data visualization were performed using the uniform manifold approximation and projection (UMAP) method within the OMIQ software environment, which was last accessed on the 15 April 2025 (from Dotmatics, Boston, MA, USA) (https://www.omiq.ai/features/flow-cytometry-software). A brief summary of the CyTOF results is provided on Supplementary Table S2.

### 4.3. Statistical Analysis

Comparisons between baseline and week 14 were performed by applying the Mann–Whitney U test. Dose groups were combined to increase statistical power. Analyses were conducted in GraphPad Prism (version 10, last) (Boston, MA, USA), which was last accessed on the 15 April 2025. Correlations were evaluated via the Spearman’s rank coefficient method.

## 5. Conclusions

Conclusively, etrasimod induces a selective redistribution of adaptive immune cells, between lymphoid tissues and the circulation in CD, providing direct mechanistic evidence of S1PR-mediated lymphocyte trafficking modulation in humans. In particular, this drug primarily retains in the LN compartment naïve and central memory T lymphocytes, with comparatively minor or no effects towards innate cells. Our data demonstrate that etrasimod reshapes immune cell distribution predominantly through altered lymphocyte egress rather than broad immunosuppression. Furthermore, our results suggest that S1PR blockade possibly influences the activation status of T helper lymphocyte populations. These findings improve our understanding of the immunological mechanisms underlying S1PR modulation in CD and pave the way for follow-up studies for further validation, with the ultimate goal to optimize the currently available therapeutic strategies in IBD.

## Figures and Tables

**Table 1:** Patient clinical, biochemical and endoscopic parameters.

Baseline Characteristics	Total (*n* = 4)
Female; *n* (%)	3 (75%)
Age at inclusion; median (range), years	44 (27–56)
Disease duration in median years (IQR)	10.1 (4.43–11.85)
*Montreal classification in CD patients*	
A1 (<16 years)	1 (25%)
A2 (17–40 years)	1 (25%)
A3 (>40 years)	2 (50%)
L1 (ileum)	1 (25%)
L2 (colon)	2 (50%)
L3 (ileocolonic)	1 (25%)
B1 (non stricturing, non-penetrating)	2 (50%)
B3 (penetrating)	2 (50%)
p (perianal disease)	2 (50%)
*Medication use in medical history*	
Biologicals (infliximab, adalimumab, vedolizumab, ustekinumab)	3 (75%)
Immunomodulators (thiopurines/methotrexate)	3 (75%)
Systemic corticosteroids (budesonide/prednisolone)	4 (100%)
Aminosalicylates	3 (75%)
*Clinical and biochemical parameters in median (IQR) at baseline*	
Crohn’s Disease Activity Index (CDAI)	219.9 (168.56–259.95)
Patient-reported Outcome (PRO-2)	16.2 (13.25–19.50)
C-reactive protein (CRP) in mg/L	3.9 (2.52–12.23)
Faecal calprotectin in mg/kg	1007.1 (203.80–1889.46)
*Endoscopic parameters*	
Total SES-CD score	7.5 (4.5–17.25)
*Etrasimod treatment arm*	
3 mg daily	3 (75%)
2 mg daily	1 (25%)
*Disease severity; n (%)*	
Moderate	3 (75%)
Severe	1 (25%)

## Data Availability

The original contributions presented in this study are included in the article/Supplementary Materials. Further inquiries can be directed to the corresponding author.

## Supplementary Materials

The supporting information can be downloaded at: https://www.mdpi.com/article/10.3390/ijms27052447/s1.

## Abbreviations

The following abbreviations are used in this manuscript:

CD	Crohn’s Disease
CDAI	Crohn’s Disease Activity Index
CRP	C-reactive protein
CyTOF	Cytometry by Time-of-Flight
DCs	Dendritic Cells
FCP	Fecal Calprotectin
HLA-DR	Human Leukocyte Antigen-DR
IBD	Inflammatory Bowel Diseases
LN	Lymph Node
LNs	Lymph Nodes
NK	Natural Killer
PB	Peripheral Blood
PBMCs	Peripheral Blood Mononuclear Cells
S1P	Sphingosine-1-Phosphate
S1PR	Sphingosine-1-Phosphate Receptor
SSA-P2	Sub-study Arm Phase 2
UC	Ulcerative Colitis
UMAP	Uniform Manifold Approximation and Projection
