# Supplementary material for: Etrasimod Treatment Modulates Circulating and Lymph Node-Derived Lymphocytes in Crohn’s Disease
Source: Int J Mol Sci. 2026 Mar 6;27(5):2447. doi: 10.3390/ijms27052447 (PMC12986399; doi:10.3390/ijms27052447)
Supplement: Supplementary file 1 [file ijms-27-02447-s001.zip › ijms-4138467-supplementary.pdf]

Effect of Etrasimod on T- Cell Subpopulations

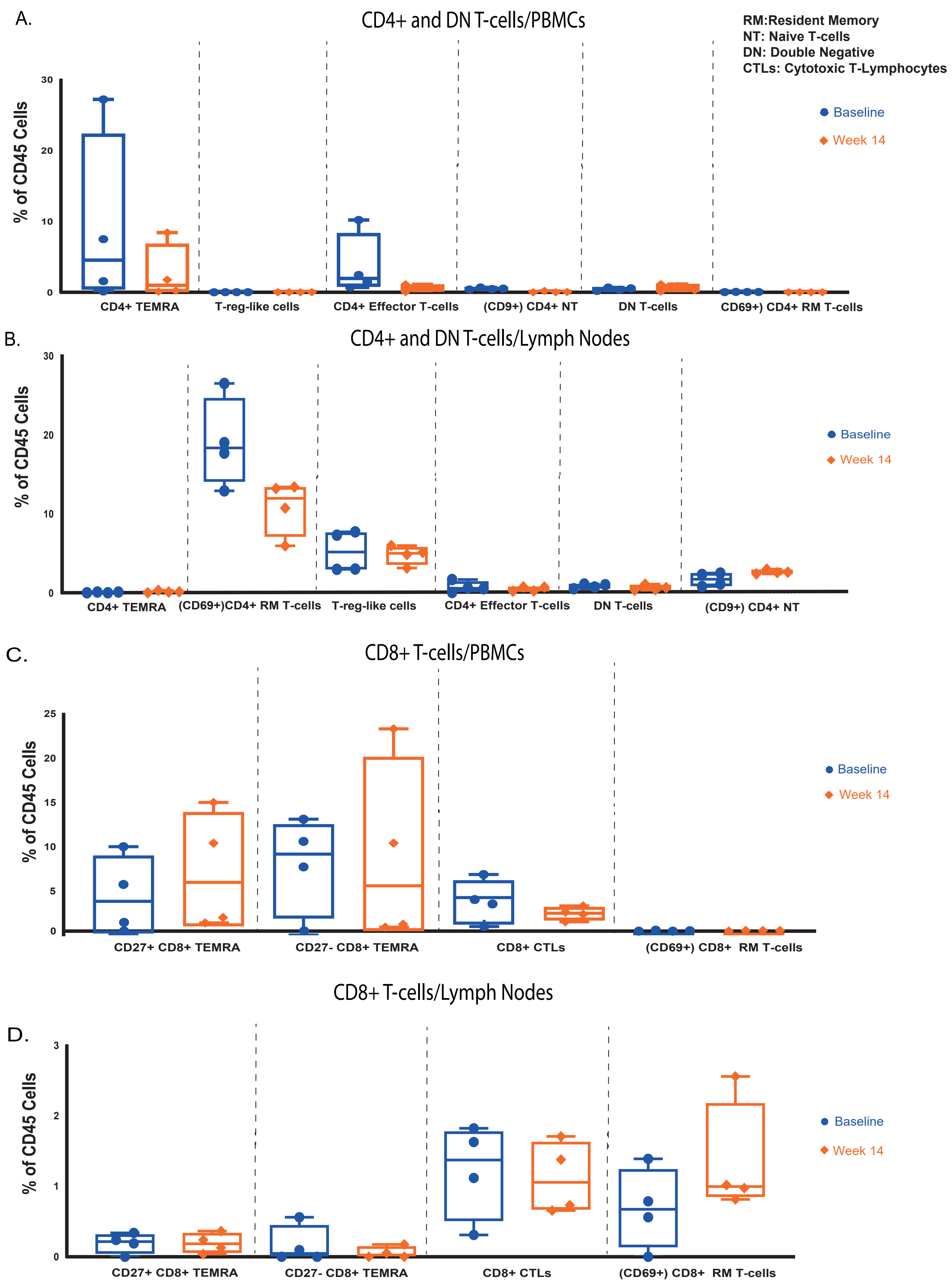

Supplementary Figure S1: A. Quantification of differences in additional CD4+ T-cell subsets and double negative T-cells between baseline and week 14 in PBMCs and **B**. in lymph nodes. **C**. Quantification of differences in additional CD8+ T-cell subsets between baseline and week 14 in PBMCs and **D.** in lymph nodes.

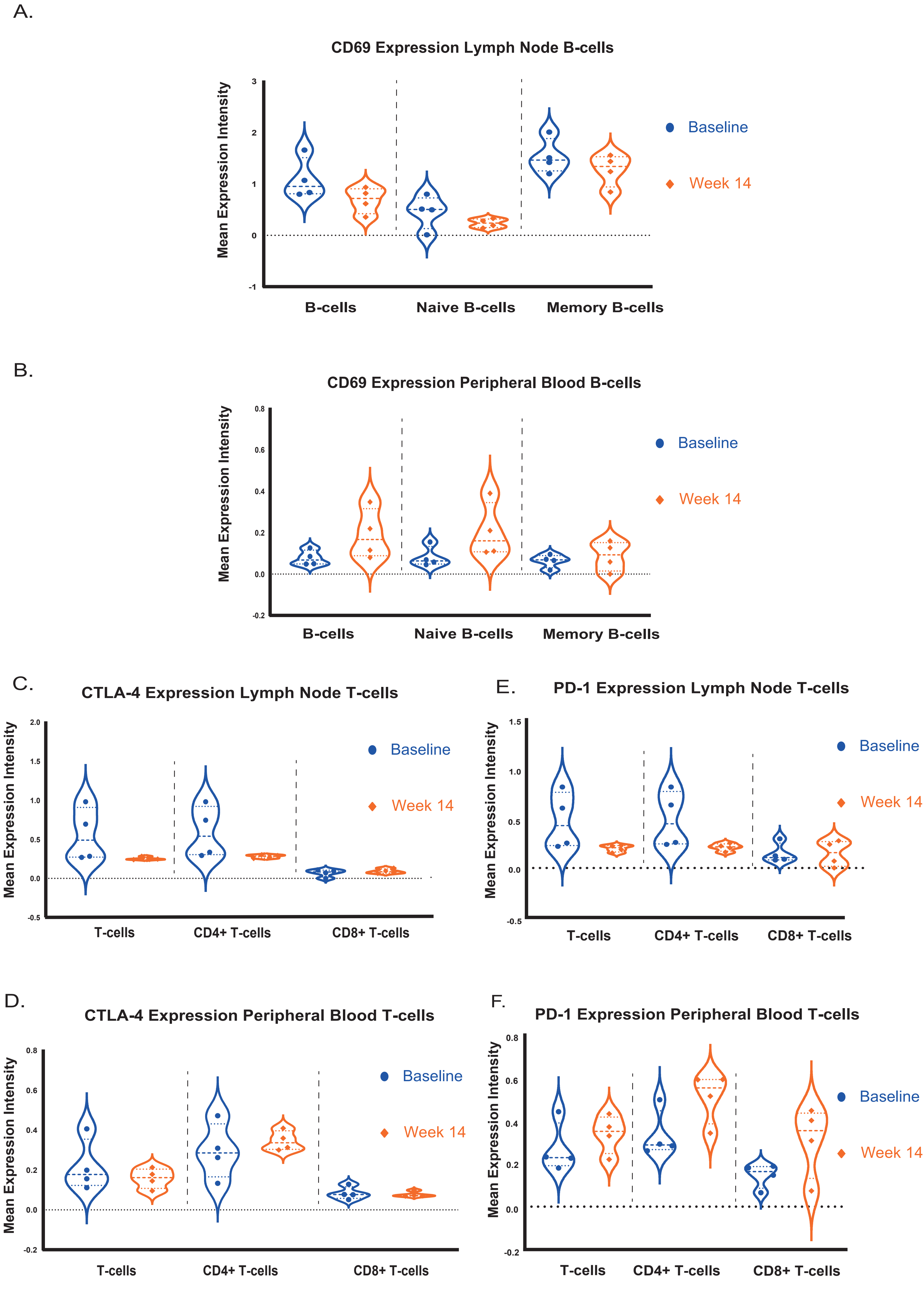
A.
CD69 Expression Lymph Node B-cells
Mean Expression Intensity
3
2
1
0
-1
B-cells
Naive B-cells
Memory B-cells
Baseline
Week 14
B.
CD69 Expression Peripheral Blood B-cells
Mean Expression Intensity
0.8
0.6
0.4
0.2
0.0
-0.2
B-cells
Naive B-cells
Memory B-cells
Baseline
Week 14
C.
CTLA-4 Expression Lymph Node T-cells
Mean Expression Intensity
2.0
1.5
1.0
0.5
0.0
-0.5
T-cells
CD4+ T-cells
CD8+ T-cells
Baseline
Week 14
E.
PD-1 Expression Lymph Node T-cells
Mean Expression Intensity
1.5
1.0
0.5
0.0
-0.5
T-cells
CD4+ T-cells
CD8+ T-cells
Baseline
Week 14
D.
CTLA-4 Expression Peripheral Blood T-cells
Mean Expression Intensity
0.8
0.6
0.4
0.2
0.0
-0.2
T-cells
CD4+ T-cells
CD8+ T-cells
Baseline
Week 14
F.
PD-1 Expression Peripheral Blood T-cells
Mean Expression Intensity
0.8
0.6
0.4
0.2
0.0
-0.2
T-cells
CD4+ T-cells
CD8+ T-cells
Baseline
Week 14

Supplementary Figure S2: A. Mean expression levels quantification of the CD69 protein marker in the B-cell subsets that were mostly affected by etrasimod, at both timepoints (baseline and week 14) in the lymph nodes and **B.** PBMCs. **C.** Mean expression levels comparison of the CTLA-4 immune checkpoint protein between the 2 timepoints, in lymph node total T-lymphocytes, CD4+ and CD8+ T-cells. CTLA-4 expression levels in lymph node total T-cells and CD4+ T- cells were reduced after 14 weeks of etrasimod treatment *(Mean Expression Intensity Difference from Baseline= -0.3 [95%CI: -0.6 to -0.02], P=0.057, and -0.31 [95%CI: 0.58 to -0.03], P=0.057, respectively)*, **D.** Mean expression levels comparison of the CTLA-4 immune checkpoint protein between the 2 timepoints, in peripheral blood total T-lymphocytes, CD4+ and CD8+ T-cells. No alterations were detected in peripheral blood. **E.** Mean expression levels comparison of the PD-1 immune checkpoint protein between the 2 timepoints, in lymph node total T-lymphocytes, CD4+ and CD8+ T-cells. In lymph node CD4+ T- cells PD-1 expression was downregulated at the week 14 timepoint *(Mean Expression Intensity Difference from Baseline= -0.28 [95%CI: -0.54 to -0.038], P=0.057)*. **F.** Mean expression levels comparison of the PD-1 immune checkpoint protein between the 2 timepoints, in peripheral blood total T-lymphocytes, CD4+ and CD8+ T-cells. In circulating CD4+ T- cells PD-1 levels were upregulated at the week 14 timepoint *(Mean Expression Intensity Difference from Baseline= +0.18 [95%CI: +0.036 to +0.3], P=0.057)*.

Supplementary Table S1: Mass cytometry (CyTOF) antibody panel.

Metal Isotope	Protein Marker	Company	Clone	Catalogue number
Y89	CD45	Standard Biotools/Fluidigm	HI30	3089003B
Pr141	CD49d	Standard Biotools/Fluidigm	9F10	3141004B
Ce142	CD19	Standard Biotools/Fluidigm	HIB19	3142001B
Nd143	CD127	Standard Biotools/Fluidigm	A019D5	3143012B
Nd144	CD11b	Standard Biotools/Fluidigm	ICRF44	3144001B
Nd145	CD68	Biolegend (self-conjugated)	Y1/82A	333802
Nd146	CD8a	Standard Biotools/Fluidigm	RPA-T8	3146001B
Sm147	CD7	Standard Biotools/Fluidigm	CD7-6B7	3144005B
Nd148	CD66a	BD Biosciences (self-conjugated)	CD66a-B1.1	551354
Sm149	CD25	Standard Biotools/Fluidigm	2A3	3149010B
Eu151	CD123	Standard Biotools/Fluidigm	6H6	3151001B
Sm152	CD141	Biolegend (self-conjugated)	1A4	344102
Eu153	CD38	BD Biosciences (self-conjugated)	HIT2	555458
Sm154	CD3	Standard Biotools/Fluidigm	UCHT1	3154003B
Gd155	PD1	Standard Biotools/Fluidigm	EH12.2H7	3155009B
Gd156	CD163	Biolegend (self-conjugated)	RM3/1	326502
Gd158	CD27	Standard Biotools/Fluidigm	L128	3158010B
Tb159	CD11c	Standard Biotools/Fluidigm	3.9	3159001B
Gd160	CD14	Standard Biotools/Fluidigm	M5E2	3160001B
Dy161	CTLA4	Standard Biotools/Fluidigm	14D3	3161004B
Dy162	CD69	Standard Biotools/Fluidigm	FN50	3162001B
Dy163	CD206	Biolegend (self-conjugated)	15-2	321102
Dy164	CD43	Biolegend (self-conjugated)	CD43-10G7	343202
Ho165	CD45RO	Standard Biotools/Fluidigm	UCHL1	3165011B
Er166	CD44	Standard Biotools/Fluidigm	BJ18	3166001B
Er167	CCR7	Standard Biotools/Fluidigm	G043H7	3167009A
Er168	PDL1	Biolegend (self-conjugated)	29E.2A3	329702
Tm169	CD33	Standard Biotools/Fluidigm	WM53	3169010B
Er170	CD45RA	Standard Biotools/Fluidigm	HI100	3170010B
Yb171	CD9	Standard Biotools/Fluidigm	SN4C3-3A2	3171009B
Yb172	CD57	Standard Biotools/Fluidigm	HCD57	3172009B
Yb173	EpCAM	Biolegend (self-conjugated)	9C4	324229
Yb174	HLA.DR	Standard Biotools/Fluidigm	L243	3174001B
Lu175	LAG.3	Standard Biotools/Fluidigm	11C3C65	3175033B
Yb176	CD4	Standard Biotools/Fluidigm	RPA-T4	3176010B
Bi209	CD16	Standard Biotools/Fluidigm	3G8	3209002B

Supplementary Table S2: Summary of the statistically significant changes in leukocyte populations after etrasimod treatment. *p-value<0.05

Cell Type	Compartment	Median % Difference from Baseline	95% Confidence Interval (CI)	p-value
**CD4+ T-cells**	PB	-22.0%	[-48.4 to -11.1]	0.03
**Total B-cells**	PB	-2.6%	[-9.5 to -0.5]	0.03
**Memory B-cells**	PB	-0.6%	[-1.3 to -0.08]	0.03
**Dendritic Cells type 1 (DCs type 1)**	PB	+10.0%	[+5.9 to +15.4]	0.03
**CD4+ Effector Memory T-cells**	PB	-2.0%	[-2.3 to -1.3]	0.03
	LN	+2.3%	[+1.6 to +11.7]	0.03
**CD4+ Central Memory T-cells**	PB	-6.0%	[-10.2 to -0.9]	0.03
	LN	+4.2%	[+0.7 to +10.1]	0.03
**CD4+ Naïve T-cells**	PB	-6.2%	[-8.9 to -3.9]	0.03
	LN	+10.7%	[+6.2 to +18.3]	0.03
**CD8+ Naïve T-cells**	PB	-2.2%	[-2.8 to -1.7]	0.03
	LN	+4.2%	[+4.0 to +4.6]	0.03
